# Felty’s syndrome

**DOI:** 10.3389/fmed.2023.1238405

**Published:** 2023-10-17

**Authors:** Christoph Wegscheider, Vera Ferincz, Karin Schöls, Andreas Maieron

**Affiliations:** ^1^Karl Landsteiner University of Health Sciences, Krems, Austria; ^2^Division of Internal Medicine, University Hospital St. Pölten, St. Pölten, Austria

**Keywords:** Felty syndrome, rheumatoid arthritis, T-LGL, rituximab, JAK inhibitor

## Abstract

Felty’s syndrome was first described in 1924 by the US-American physician Augustus Roi Felty as a triad of rheumatoid arthritis, splenomegaly and leucopenia. Even nearly 100 years later, this rare syndrome is still paralleled by diagnostic and therapeutic challenges and its pathogenesis is incompletely understood. Neutropenia with potentially life-threatening infections is the main problem and several pathomechanisms like Fas-mediated apoptosis, anti-neutrophil antibodies, anti-G-CSF antibodies, neutrophil consumption in the context of NETosis and suppression of granulopoiesis by T-LGLs have been suggested. Felty’s syndrome has various differential diagnoses as splenomegaly and cytopenia are common features of different infectious diseases, malignancies and autoimmune disorders. Additionally, benign clonal T-/NK-LGL lymphocytosis is increasingly noticed in Felty’s syndrome, which further complicates diagnosis. Today’s treatment options are still sparse and are largely based on case reports and small case series. Methotrexate is the mainstay of therapy, followed by rituximab, but there is less evidence for alternatives in the case of adverse reactions or failure of these drugs. This article gives an updated review about Felty’s syndrome including its pathogenesis and treatment options.

## Introduction

Felty’s syndrome (FS) was first described in 1924 by the US-American physician Augustus Roi Felty as a combination of rheumatoid arthritis (RA), splenomegaly and leucopenia. Even nearly 100 years later, this syndrome is still paralleled by diagnostic and therapeutic challenges. Therefore, current knowledge regarding pathogenesis, diagnosis and treatment is summarized in the following sections.

## Pathogenesis

### Felty’s syndrome and T-LGL leukemia—a common disease spectrum?

The pathogenesis of Felty’s syndrome is still incompletely understood. Some studies suggested a linkage with T-LGL (T-large granular lymphocyte) leukemia, as the clinical picture with neutropenia, splenomegaly and rheumatoid arthritis can be similar in both diseases, making differential diagnosis difficult. As explained in detail later, T-LGL leukemia is characterized by monoclonal expansion of T-LGLs (mostly CD8^+^), which usually comprise only 10–15% of mononuclear blood cells in healthy individuals (1). Interestingly, expansion of T-LGLs is present in many patients with Felty’s syndrome as well, although mostly poly−/oligoclonal. In one study comprising 21 patients with T-LGL lymphocytosis, 9 patients (=43%) suffered from seropositive rheumatoid arthritis, splenomegaly and neutropenia and were therefore regarded as having Felty’s syndrome. After assessing clonality in those patients using T-cell receptor studies, 3 patients fulfilled the WHO (world health organization) criteria for T-LGL leukemia. Another 3 patients also had monoclonal T-LGLs, but T-LGL count was <2 × 10^9^/L and 2 patients had polyclonal expansion of T-LGLs, which is a common finding in autoimmune diseases. Interestingly, there was a mixed T-LGL composition in 1 patient, showing polyclonal as well as monoclonal T-LGLs simultaneously, which was interpreted as a possible transition state (2). But whether the T-LGL lymphocytosis that accompanies some cases of Felty’s syndrome can degenerate into T-LGL leukemia is unknown so far. Consecutive clonality studies in patients with Felty’s syndrome might be useful to examine if such a transition is possible and whether it can be influenced by immunosuppressive treatment.

A major driver of clonal T-LGL expansion in T-LGL leukemia is dysregulated JAK/STAT signaling, caused by somatic gain-of-function mutations in STAT3 (signal transducer and activator of transcription 3). STAT3 acts as a transcription factor with anti-apoptotic as well as proliferative effects and additionally, its activation in T-LGL leukemia patients correlates with FasL (Fas ligand) levels, explaining the occurrence of neutropenia in these patients (3). STAT3 mutations were noted in 30–70% of T-LGL leukemia cases and although not pathognomonic for this disease, the detection of STAT3 mutations can aid in T-LGL leukemia diagnosis in an appropriate clinical context. Savola et al. reported STAT3 mutations in 40% of FS patients as well (4), leading to speculations about a common pathogenesis of T-LGL leukemia and Felty’s syndrome. However, a recent study found STAT3 mutations in only 10% of FS cases (5). In this study, T-LGL clonality was more thoroughly assessed to allow a better distinction between FS cases and patients with T-LGL leukemia. The FS population studied by Savola et al. was heterogenous though, possibly leading to a higher STAT3 mutation detection rate because of admixed T-LGL leukemia cases.

Another similarity of Felty’s syndrome and T-LGL leukemia was suggested by older studies showing a frequent expression of HLA-DRB1 alleles, especially HLA-DRB1*04:01 and HLA-DRB1*04:04 (HLA = human leukocyte antigen). HLA-DRB1 was expressed in 90% of cases with Felty’s syndrome as well as in patients with T-LGL leukemia in the wake of rheumatoid arthritis. By contrast, there was no significant HLA-association in patients with T-LGL leukemia without accompanying rheumatoid arthritis (6). However, as the studied FS populations at that time likely contained T-LGL leukemia patients as well due to insufficient clonality assessments, those findings should be interpreted with caution.

Taken together, it is yet unclear whether Felty’s syndrome and T-LGL leukemia form a common disease spectrum and many studies were based on heterogenous populations, hampering interpretation.

### Possible role of NETs in autoimmunity and neutropenia

The secretion of neutrophil extracellular traps (NETs) is another defense mechanism of neutrophils alongside phagocytosis and degranulation of antimicrobial granules. NETs have recently attracted attention in autoimmunity research and they might play an important role in the pathogenesis of Felty’s syndrome as well. The exact mechanisms of NET formation are still unknown, but a variety of pathogens (bacteria, viruses, fungi, protozoa), cytokines [e.g., tumor necrosis factor α (TNFα), interleukin 8 (IL-8), interferon α (IFNα)] and chemical noxae (e.g., hydrogen peroxide, nitric oxide) are able to trigger NET formation in neutrophils. During this process, enzymes like MPO (myeloperoxidase) and NE (neutrophil elastase) translocate into the nucleus and break down histones, leading to chromatin decondensation. The chromatin then passes the damaged nuclear membrane and enters the cytoplasm, where it encounters various proteins. A web consisting of DNA (deoxyribonucleic acid), antimicrobial enzymes (e.g., proteinase 3, cathepsin G, myeloperoxidase) and other antimicrobial agents (e.g., lactoferrin, cathelicidin) is finally released into the extracellular space. The negatively charged DNA and the various positively charged proteins attached upon it allow for numerous non-covalent interactions with the surface of pathogens, which are literally trapped and killed in those nets. It is thought that NET formation comes at the expense of neutrophil death and this process was termed “NETosis “by analogy with other forms of cell death. But subsequently, NET formation was detected in living, albeit anuclear neutrophils as well (7), which might then be removed by macrophages. Overall, NET formation thus comes along with neutrophil consumption.

Additionally to their antimicrobial effects, NETs might play an important role in the development of autoimmune diseases too, as their secretion into the extracellular space expose various intracellular antigens, some of which are well known in different diseases (e.g., DNA, MPO, proteinase 3) (8). The pathogenetic importance of NETs is best known in SLE (systemic lupus erythematosus), wherein NETs not only represent a source of antigens but also have cytotoxic effects on endothelial cells and therefore broader pathogenetic implications.

During NET formation, histones can undergo post-translational modifications (e.g., acetylation, methylation, citrullination) (9) and ACPAs (anti-citrullinated protein antibodies) in RA patients reacted with citrullinated and even with acetylated histones in NETs (10). Similarly, patients with Felty’s syndrome had antibodies against NET components including citrullinated histones (11). Therefore, it is tempting to postulate a pathogenetic role of NET formation and NETosis in Felty’s syndrome and FS-associated neutropenia. However, additional pathophysiological studies are necessary in this regard and as only a minor subset of RA patients develops Felty’s syndrome, the presumptive role of NETs in Felty’s syndrome pathogenesis can only be a partial one.

### Other causes of neutropenia

The emergence of neutropenia in Felty’s syndrome seems to be multifactorial and supposed mechanisms include increased peripheral destruction as well as suppressed production in the bone marrow. Antibodies against neutrophil antigens that lead to complement activation, opsonization and consecutive phagocytosis by macrophages in the spleen and bone marrow is a well-accepted mechanism causing primary autoimmune neutropenia (AIN). In AIN, the autoantibodies act against surface proteins like Fc-gamma receptor IIIb or CD11b (an integrin) on neutrophils (12). However, much less is known about the neutrophil antigens responsible for neutropenia in Felty’s syndrome. Ditzel et al. identified autoantibodies against eukaryotic elongation factor 1A-1 (eEF1A-1) (13), but whether these antibodies cause neutropenia is unknown to date. Van Gaalen et al. postulated, that antibodies against glucose-6-phosphate isomerase (G6PI-Ab) might contribute to neutropenia via forming of G6PI-Ab containing immune complexes on vessel walls, leading to margination of neutrophils (14). However, as later studies detected G6PI-Ab not only in FS patients, but also in other rheumatic diseases that are not associated with neutropenia (e.g., gout, spondyloarthritis) (15), the role of G6PI-Ab in Felty’s syndrome remains elusive.

Another cause of neutropenia in Felty’s syndrome might be Fas-mediated apoptosis. Fas receptor (Fas, CD95) and Fas ligand (FasL, CD95L) both exist as membrane-bound (mFas and mFasL respectively) as well as soluble forms (sFas and sFasL respectively). Interaction between Fas and FasL can have different impacts on the cells involved, from which apoptosis of the Fas-expressing cell is best known. FasL is expressed by cytotoxic T-cells, which are thus able to kill Fas-expressing cells (e.g., antigen-presenting cells like dendritic cells or neutrophils). Because T-cells express both Fas and FasL following their activation, the Fas/FasL pathway is also important for preventing an overshooting T-cell response. This process called activation-induced cell death (AICP) might play a crucial role in immune regulation and autoimmunity prevention.

Studies with patients suffering from rheumatoid arthritis or T-LGL leukemia found elevated sFasL serum levels (16, 17) and at least in T-LGL leukemia patients, sFasL-mediated apoptosis of neutrophils was demonstrated *in vitro* (16). Interestingly, the Fas/FasL pathway can also have an anti-apoptotic effect. In T-LGL leukemia patients, Fas/FasL-mediated apoptosis of leukemic cells was blocked. This might be due to high serum levels of sFas found in this study, which occupies FasL and thus prevents its interaction with Fas, rendering leukemic cells resistant to apoptosis (18).

Additionally, antibodies against G-CSF (granulocyte colony-stimulating factor) might play a role in neutropenia. A study with 15 Felty’s syndrome patients detected anti-G-CSF IgG antibodies in 11 cases. At least in 3 from 9 anti-G-CSF positive cases (=33%), these antibodies had a neutralizing effect on G-CSF too. But because G-CSF activity was unaffected in the remaining study participants, additional mechanisms like a reduced responsivity of neutrophils to G-CSF was suggested. Interestingly, anti-neutrophil antibodies were detected as well in all 7 patients tested who were anti-G-CSF positive (19).

In 1983, bone marrow-directed mechanisms in 27 FS patients were investigated in one study. Cell cultures showed less colony forming units (CFU) compared to controls. In 7 patients, bone marrow suppression was attributed to CD8^+^ T-cells and in 5 patients, an unknown serum inhibitory factor led to decreased CFUs. Feeder layers from another 5 patients failed to induce CFUs in control’s bone marrow cultures, which was suggested to be caused by a lack of colony stimulating factor (CSF) generation in FS patients (20). Again, it should be recognized that these results might be confounded by a heterogeneous study population (i.e., possibly admixed T-LGL leukemia cases) and newer studies would be useful as a proof. Whether the CD8^+^ T-cells in the above study represented T-LGLs is unknown, but T-LGLs were shown to suppress granulocyte-macrophage progenitor cells *in vitro* (21) although there is still uncertainty about how this suppression is mediated (i.e., via cell–cell interaction or via cytokines).

### Causes of pancytopenia in Felty’s syndrome

There are various presumed pathomechanisms for neutropenia (i.e., increased neutrophil consumption in the context of NETosis, Fas-mediated apoptosis, suppression of granulopoiesis by T-LGLs, anti-neutrophil antibodies, anti-G-CSF antibodies), but Felty’s syndrome as well as T-LGL leukemia can sometimes present with pancytopenia, whose causes are less well understood. One possible explanation for pancytopenia is an increased sequestration of blood cells in the spleen due to splenomegaly. Histopathological studies examining spleens from patients with Felty’s syndrome identified unspecific changes like an enlarged red pulp, plasmacytosis and hyperplastic lymph follicles as the histological correlate of splenomegaly (22). In T-LGL leukemia, the red pulp is expanded as well, but additionally the spleen is infiltrated by monoclonal T-cells (23). Strikingly, splenic size does not correlate with the extent of cytopenia (24, 25) and splenectomy was found to only partially or transiently revert cytopenia (24, 25). These findings suggest that hypersplenism cannot be the sole reason for pancytopenia. It is likely that T-LGLs account for anemia and thrombopenia in a similar way as they do in neutropenia. In the 1990s, T-LGLs were found to be able to lyse erythroblasts via interaction between MHC I (major histocompatibility complex) molecules and KIR receptors (killer cell inhibitory receptor) (26). KIR receptors are actually a characteristic feature of natural killer (NK) cells but are expressed on T-LGLs as well. They interact with MHC I molecules (HLA-A, HLA-B, HLA-C) and can be divided into activating and inhibitory KIR receptors according to their molecular structure. Overall, KIR receptors facilitate the detection of abnormal cells like infected or neoplastic cells, which exhibit an aberrant HLA expression (i.e., “missing self “hypothesis). It was found that each KIR receptor has a distinct HLA ligand (e.g., the ligand for KIR 2DL1 is HLA-C2), which is called a “KIR/HLA match” (27). Nowakowski et al. studied the KIR receptor phenotype as well as the HLA I genotype in 7 patients with T-LGL leukemia. In 5 patients, there was a KIR/HLA mismatch, which means that the HLA I molecule could not be identified by the KIR receptor. All these patients had pancytopenia, whereas the remaining patients with KIR/HLA match had a normal blood count (28). Because clonal T-LGLs usually express just one KIR isoform (or rarely two), it is conceivable that the resulting KIR/HLA mismatch leads to lysis of hematopoietic progenitor cells and pancytopenia. Even T-CUS (T-cell clonopathy of unknown significance) as well as polyclonal T-LGL expansion were linked with the suppression of hematopoiesis (29, 30). As mentioned earlier, Felty’s syndrome is often associated with T-LGL expansion, suggesting its role in cytopenia. However, causation still needs to be proven through studies involving patients with Felty’s syndrome.

### Neutrophil dysfunction

Older studies conducted by Ruderman et al. and Sienknecht et al. already demonstrated that there is no correlation between the extent of leucopenia and the number of infections in patients with Felty’s syndrome (24, 25). Strikingly, even raising neutrophil counts by splenectomy did not alter infection rate significantly (24), suggesting the contribution of neutrophil dysfunction. Subsequently, studies found an impaired phagocytosis of opsonized bacteria, which was attributed to a diminished expression of Fc receptors on neutrophils. This reduction of Fc receptors is thought to be due to circulating immune complexes that bind to Fc receptors, causing their internalization (31).

Studies conducted in the 1970s and 1980s also demonstrated impaired chemotaxis (32, 33) as well as decreased superoxide production (34). Neither of these findings was examined in more recent studies.

## Clinical manifestations

Felty’s syndrome typically consists of the triad of rheumatoid arthritis, neutropenia and splenomegaly. Rheumatoid arthritis in Felty’s syndrome is usually seropositive and severe, leading to erosions and joint deformities. This had been revealed in older studies as well as in a more recent cohort study, which found joint erosions in 77% of Felty’s syndrome patients at the time of diagnosis (35). In this cohort study, 92 and 96% of cases tested positive for rheumatoid factor and ACPA, respectively (35). Different studies also demonstrated ANA (antinuclear antibodies) positivity in 55–84% of cases (24, 25, 36, 37) and in 6% of patients, antibodies against double-stranded DNA were detected (36). Usually, joint disease precedes the emergence of neutropenia and splenomegaly by several years (11–16 years on average) (36, 37). Only in isolated cases, neutropenia and splenomegaly are diagnosed before or at the onset of rheumatoid arthritis (35, 38). When compared to rheumatoid arthritis without accompanying Felty’s-Syndrome, Felty’s syndrome is more often paralleled by extraarticular manifestations of RA, like rheumatoid nodules (71–82% of cases), vasculitis (24–28%), pleuritis (15–22%), episcleritis (3–11%), and pericarditis (7%). Skin ulcers, mostly at the legs, are noted in 78% of cases (24, 36, 37). These ulcers have a multifactorial etiology, including common causes like chronic venous insufficiency and atherosclerosis as well as vasculitis and ulcerated rheumatoid nodules (39, 40). Skin ulcers often get contaminated and their occurrence correlates with infection risk (40). Other clinical manifestations of Felty’s syndrome include lymphadenopathy (16–42% of cases) and yellowish/brown skin pigmentations (24, 25, 36, 37).

A prerequisite for diagnosis is neutropenia, defined as an absolute neutrophil count (ANC) below the reference range of the respective laboratory (in most laboratories, neutropenia is defined as ANC < 1,500/μL). About half of Felty’s syndrome patients present with severe neutropenia (ANC < 500/μL) and even agranulocytosis (ANC < 100/μL) is possible (35). Neutropenia increases the infection risk, with skin infections and respiratory infections being most frequent. Common Gram-negative and Gram-positive bacteria like *Staphylococcus aureus*, *Haemophilus influenzae*, *Pseudomonas aeruginosa* and *Streptococcus* spp. are involved. Left untreated, infection might proceed to sepsis and death, making the increase in neutrophil counts a main goal of therapy.

Additionally to neutropenia, studies also reported varying rates of thrombopenia (15–48% of cases) and anemia (79–100%) (24, 25, 36). As mentioned above, hypersplenism and myelosuppression are possible reasons for pancytopenia. Anemia can also be caused by chronic inflammation/active rheumatoid arthritis or by blood loss due to bleeding esophageal varices.

Nowadays, splenomegaly is no longer a prerequisite for the diagnosis of Felty’s syndrome, but most patients will show some degree of splenomegaly. Sienknecht et al. for example reported splenic weights up to 1,650 g (normal spleen weight: 150–200 g) (24).

Like other rheumatic diseases, Felty’s syndrome can be accompanied by nodular regenerative hyperplasia (NRH) of the liver, leading to non-cirrhotic portal hypertension. NRH is characterized by multiple small (<3 mm) regenerative nodules and only minimal fibrosis, as opposed to liver cirrhosis. NRH is thought to be caused by immune-mediated damage of small portal vein endothelium with consecutive hypoperfusion and apoptosis of hepatocytes. During the regeneration process, growth factors cause hyperplasia of adjacent hepatocytes, resulting in multiple nodules (41). In contrast to liver cirrhosis, liver function is preserved, but portal hypertension and its consequences (i.e., ascites, splenomegaly, esophageal varices) can develop similarly in NRH. Besides NRH, splenomegaly can contribute to portal hypertension as well due to increased blood flow in the portal venous system. Taken together, portal hypertension in Felty’s syndrome can be of intra-and prehepatic origin. The most serious sequel of portal hypertension is bleeding from esophageal varices, which occurred in some patients with Felty’s syndrome (42) and which is why endoscopic screening for varices can be recommended.

Like rheumatoid arthritis, Felty’s syndrome is associated with an increased risk of malignancy. While there is a 2.5-fold higher lymphoma risk in RA when compared to the general population (43), this risk is even higher (5-fold) in Felty’s syndrome, as shown in a Swedish registry study involving 952 patients with Felty’s syndrome (44). Generally, it seems that malignancy risk correlates with the extent of inflammatory (i.e., rheumatoid arthritis) activity. Baecklund et al. studied the incidence of lymphomas taking account of RA activity and detected a 8-fold and 70-fold higher incidence for “moderate “and “high “RA activity, respectively, compared to patients with “low“RA activity (45). It is believed that the chronic immunostimulation facilitates clonal selection and finally malignant transformation of lymphocytes. Felty’s syndrome often represents a severe and difficult-to-treat form of rheumatoid arthritis, by which its higher lymphoma incidence, when compared to isolated RA, might be explained. Additionally, RA patients have a 1.6-fold higher risk for lung cancer (43), which is at least partially attributed to chronic inflammation too. On the other hand, the risk for colorectal cancer, breast cancer, cervical cancer, prostate cancer as well as melanoma in RA patients does not seem to be significantly increased compared to the general population (43). This might be similar in patients with Felty’s syndrome.

## Epidemiology

The prevalence of Felty’s syndrome declined in the last decades from 1.5% in 1985 to currently 0.5%. This might be partially due to new classification criteria for LGL leukemia established in 1985. Before then, some cases of LGL leukemia might have been classified as Felty’s syndrome. Additionally, improved treatment options for rheumatoid arthritis since 1985 (e.g., methotrexate) might also play a role (46, 47). Felty’s syndrome is usually diagnosed between the fourth and sixth decade of life and women are affected in 60–85% of cases (24, 25, 35–37). Family history for rheumatoid arthritis is positive in approximately 40% of FS patients and HLA-DRB1 alleles are identified in up to 90% of FS cases (36). Frequencies of these risk alleles vary geographically, corresponding to the prevalence of rheumatoid arthritis and Felty’s syndrome. Thus, RA prevalence is higher in Western Europe and North America than in Asia or Africa (48). Accordingly, the frequency of HLA-DRB1*04:01 for example is 7% in Finland, but much lower in Koreans (0.7%) and Afro-Americans (2.6%) (49–51).

## Differential diagnosis

The differential diagnosis of Felty’s syndrome includes various conditions presenting with cytopenia and/or splenomegaly (Supplementary Tables S1, S2). Even its typical triad of rheumatoid arthritis, splenomegaly and neutropenia does not allow a straightforward diagnosis, as RA might just be a coincidence. Therefore, it is essential to rule out malignant diseases, which usually requires bone marrow biopsy. Some hematological conditions and diseases that resemble Felty’s syndrome might be a bit confusing (Supplementary Table S3) and are therefore highlighted in the upcoming section.

### T-LGL and NK-LGL disorders

Large granular lymphocytes usually comprise 10–15% of peripheral blood mononuclear cells. Most of them are T-cells (T-LGL) and only 15% represent NK-cells (NK-LGL). Both subsets can undergo malignant transformation, causing T-LGL and aggressive NK-LGL leukemia, respectively. T-LGL leukemia is an indolent disease which only requires treatment in the case of symptomatic cytopenia (usually neutropenia). Aggressive NK-LGL leukemia represents the exact opposite, with a median survival of <2 months (52). Many cases of T-LGL leukemia emerge in the wake of autoimmune diseases, thereby suggesting chronic (auto)antigen stimulus with consecutive clonal LGL selection as the underlying pathomechanism. This hypothesis is supported by the fact that LGLs typically exhibit a memory phenotype, thus must have encountered an antigen. Up to 25% of T-LGL leukemia cases occur in patients suffering from rheumatoid arthritis (53) and T-LGL leukemia is thus an important differential diagnosis of Felty’s syndrome. In T-LGL leukemia, neutropenia is seen in up to 84% of cases and studies also reported significant rates of anemia (24–89%) and thrombopenia (19–36%) (54). In contrast to Felty’s syndrome, men and women are equally affected in T-LGL leukemia and splenomegaly only occurs in 19–50% of cases (54), whereas its absence in Felty’s syndrome is exceptional. Unlike one might suggest from the term “leukemia“, lymphocytosis (absolute lymphocyte count >4 × 10^9^/μL) is present in only 50% of cases and normal lymphocyte counts therefore do not exclude T-LGL leukemia. B symptoms and lymphadenopathy occur infrequently in T-LGL leukemia.

Using flow cytometry from peripheral blood or bone marrow specimen, LGLs can be quantified and up to 0.25 × 10^9^/L cells are regarded normal in healthy individuals, although there is some disagreement concerning this upper limit of normal. The WHO defines T-LGL leukemia as a persistent (>6 months) increase in T-LGL counts, usually to 2–20 × 10^9^/L, without a clearly identified cause (52). However, it is common practice to consider even T-LGL counts <2 × 10^9^/L as sufficient for T-LGL leukemia diagnosis, if both monoclonality and an appropriate clinical picture (e.g., cytopenia, recurrent infections, splenomegaly, accompanying autoimmune disease) are present (55).

On the other hand, monoclonal T-LGL counts <2 × 10^9^/L in the absence of symptoms are insufficient for the diagnosis of T-LGL leukemia and this constellation is often called T-CUS. In a case series involving 30 patients with T-CUS, clinical course remained indolent without any intervention in 80% during the follow-up period of 9 years, but some T-CUS cases developed hemolytic anemia, immune thrombocytopenia or transformed into T-LGL leukemia necessitating treatment (29).

Most T-LGLs express CD8 like cytotoxic T-cells. Additionally, these cells usually exhibit a phenotype akin to terminal effector memory T-cells (i.e., CD45RA^+^ CD62L^−^) and express certain NK-cell markers (CD57, CD16) as well as the α/β T-cell receptor (α/β TCR). Uncommon variants include CD4^+^ T-LGLs with α/β TCR and CD8^+^ or CD4^−^CD8-T-LGLs with γ/δ T-cell receptor.

Using PCR (polymerase chain reaction)-based T-cell receptor gene rearrangement studies or flow cytometric TCR-phenotyping, T-LGLs can be assessed for clonality. If there are not enough T-LGLs in the peripheral blood for analysis, bone marrow biopsy must be performed to get more sample material. In doing so, bone marrow can be concurrently assessed for infiltration by leukemic cells, which is usually present in T-LGL leukemia (52).

In contrast to T-LGL leukemia, aggressive NK-LGL leukemia can usually be distinguished from Felty’s syndrome by its clinical course alone. Delineation of NK-LGLs can be done by flow cytometry (most frequent phenotype: CD3^−^CD4^−^CD8^−^CD16^+^CD56^+^CD57^−^) (52). Another difference to T-LGL leukemia is the lack of associated autoimmune disorders in aggressive NK-LGL leukemia.

Besides T-LGL leukemia and aggressive NK-LGL leukemia, there exists a third monoclonal LGL disorder termed CLPD-NK (chronic lymphoproliferative disorder of NK cells) by the WHO. CLPD-NK is characterized by persistently (>6 months) increased NK-LGL counts (usually >2 × 10^9^/L) without a clearly identified cause and like T-LGL leukemia, it is a mostly indolent condition, which sets it apart from aggressive NK-LGL leukemia (52).

### Reactive LGL lymphocytosis

Various autoimmune disorders (e.g., rheumatoid arthritis, Felty’s syndrome, vasculitides, SLE), viral infections (e.g., EBV, CMV, HIV) and hematologic diseases (e.g., myelodysplastic syndromes, aplastic anemia, myeloproliferative neoplasms) can be paralleled by a clonal expansion of T-LGLs and/or NK-LGLs. LGL expansion was also observed following transplantation of solid organs or hematopoietic stem cells and in the context of immunomodulatory therapies (e.g., dasatinib, rituximab) (52, 56, 57). In the case of viral infections and autoimmune diseases, chronic antigen stimulation with subsequent clonal selection is thought to be the underlying pathomechanisms of LGL lymphocytosis. Contrary to this, in hematologic diseases like myelodysplastic syndromes or aplastic anemia, it is less clear if LGL lymphocytosis really represents a “reactive “phenomenon, or is actually a part of the underlying pathology (58).

LGL lymphocytosis is usually comprised of poly-or oligoclonal LGLs, which sets it apart from monoclonal LGL leukemia. Unfortunately, clonality assessment cannot differentiate all cases of LGL lymphocytosis from LGL leukemia because some viral infections have been linked with monoclonal LGL expansion too (59, 60). But at least in CMV infection, this monoclonal LGL lymphocytosis appears to be a transient phenomenon (59). Nevertheless, there are cases of LGL leukemia in HIV-infected patients (61), which is why laboratory monitoring should be performed regularly to detect transition of an allegedly benign monoclonal LGL lymphocytosis into LGL leukemia.

### Hepatosplenic T-cell lymphoma

HSTL is a rare and aggressive extranodal T-cell lymphoma with a median survival of less than 2 years. Median age at diagnosis is 35 years and men are affected more frequently (9-fold) than women. Approximately 20% of HSTL cases occur in the wake of chronic immunosuppression or chronic antigen stimulation (52, 62). Thus, HSTL was observed in patients following organ transplantation or chemotherapy as well as in the context of immunosuppressive treatment of inflammatory bowel disease or rheumatic disorders. Especially the use of thiopurines (azathioprine, 6-mercaptopurin) over a course of >2 years was linked to the emergence of HSTL, but some cases were also noted following exclusive TNFα inhibitor exposure. However, it is unknown whether the occurrence of HSTL is caused by immunosuppressive agents or by the underlying autoimmune disorder/immune dysregulation itself. Similar to LGL leukemia, chronic (auto)antigen stimulation with subsequent clonal selection is thought to be underlying pathomechanism of HSTL (63). HSTL is characterized by the occurrence of mature T-cells, which infiltrate spleen, liver and bone marrow, leading to hepatosplenomegaly and (pan)cytopenia (64). At first glance, confusion with Felty’s syndrome is thus possible but differentiation is usually easily accomplished using flow cytometric immunophenotyping. HSTL typically features CD4/CD8 negative T-cells expressing the γ/δ T-cell receptor subtype, whereas T-LGL leukemia is usually characterized by CD8^+^ T-cells with α/β T-cell receptor. However, it should be kept in mind that akin to T-LGL leukemia, there exist HSTL variants, that express the α/β T-cell receptor (52).

### Hairy cell leukemia

HCL is a rare B-cell neoplasm that predominantly affects middle-aged to elderly adults and has a male preponderance. HCL is thought to arise from an activated memory B-cell that has acquired an activating gene mutation in BRAF, which leads to constitutive activation of the RAF-MEK-ERK signaling pathway (MEK: mitogen-activated protein kinase kinase, ERK: extracellular signal-regulated kinase). This results in enhanced cell survival and proliferation and is also the presumed cause for the cytoplasmic projections that give those cells their “hairy” morphology. The tumor cells predominantly infiltrate bone marrow and spleen, causing pancytopenia and splenomegaly. Occasionally, HCL can lead to PAN-like vasculitis (PAN: Polyarteritis nodosa), rendering clinical differentiation from Felty’s syndrome especially difficult. HCL diagnosis is made by bone marrow biopsy showing a distinctive cytomorphology in conjunction with immunophenotyping (52).

## Treatment

When interpretating older studies, it should always be kept in mind that the study populations at that time were likely heterogeneous, containing FS patients as well as patients with T-LGL leukemia, because flow cytometric immunophenotyping and clonality studies were not available. This might have influenced treatment outcomes. The main treatment goal in Felty’s syndrome is the improvement of neutropenia and infection rate. Initially, splenectomy was the only treatment option in the absence of efficient pharmacotherapy. However, splenectomy regularly results in only transient increases of neutrophils. A sustained normalization of neutrophil counts over a period exceeding 6 months can be achieved in approximately 80% of cases (65), but this percentage often declines steadily over time [e.g., one study reported normal neutrophil counts in 62% of patients after 5 years follow-up (36)]. Infection rate is often not controlled by splenectomy either and roughly one third of patients continue to suffer from recurrent infections (65). Interestingly, there is no correlation between infection rate and neutrophil count. Besides neutropenia, splenectomy usually improves anemia and thrombopenia as well, although these manifestations are seldom severe enough to be clinically relevant, let alone necessitating splenectomy. Apart from its hematological ramifications, splenectomy also has beneficial effects on the healing of leg ulcers (37). Overall, splenectomy has become less important over time and today, its indication is mainly limited to recurrent infections despite medical therapies.

As the occurrence of circulating immune complexes in Felty’s syndrome had been recognized early and had been attributed pathogenetic importance, there were several attempts to remove those immune complexes using plasmapheresis. But studies showed conflicting results (66), which is why plasmapheresis currently has no role in the treatment of Felty’s syndrome. This is also true for intravenous immune globulin (IVIG) therapy, which failed to improve neutrophil counts significantly in most studies (67).

During the last decades, various agents including lithium, ACTH (adrenocorticotropic hormone), parenteral gold and D-penicillamine were tried as conservative treatment in Felty’s syndrome, but neither of them prevailed because of either inefficacy or intractable adverse reactions. In 1980, methotrexate was used in patients with Felty’s syndrome for the first time and it still represents first-line treatment. But even for methotrexate, evidence is limited to case reports and some case series. In a case series involving 7 patients for example, methotrexate significantly increased mean neutrophil count from 1.95 × 10^9^/L to 3.92 × 10^9^/L within 1 year. Neutrophil counts already started to rise after 4 weeks of treatment (68). Additionally, studies also reported a decline in infection rate (69) and beneficial effects on the healing of vasculitis-mediated ulcers (69). The efficacious methotrexate dose in most case reports and case series was 7.5 mg, which is very low when compared to usual doses used nowadays in rheumatology. Due to limited data, frequencies of treatment success and treatment failure are unknown. One case report actually reported a further decline in neutrophil count due to methotrexate (70), highlighting the importance of regular laboratory monitoring during therapy.

If there is no adequate increase in neutrophil count under methotrexate therapy, the next step is to add rituximab (71–75). The pathogenetic considerations underpinning its choice implicate the presumed role of antibodies (e.g., ACPA, anti-neutrophil antibodies, anti-G-CSF antibodies) and B-cells in Felty’s syndrome. B-cells interact with T-cells via cytokines and antigen presentation and B-cell depletion using rituximab might thus have beneficial effects in Felty’s syndrome as well. A systematic review of case reports revealed normalization of neutrophil counts in 62.5% of cases after the first rituximab course. In most cases, this was paralleled by declining infection rates too (76). Usually, rituximab is administered as an intravenous infusion of 1,000 mg, repeated once 2 weeks later. But case reports also showed efficacy for the rituximab regime used in vasculitis treatment (i.e., weekly doses of 375 mg/m^2^ body surface area for 4 doses) (76). Depending on RA activity and neutrophil count, repeated rituximab courses may be needed. To date, long-term data concerning rituximab maintenance therapy in Felty’s syndrome is sparse, but Brockbank et al. reported sustained remission in one patient after 6 years of follow-up (74). However, some reports also pointed out rituximab-refractory cases, in which RA activity declined after 6 months of treatment, but without improving neutrophil count or infection rate (77). But it should be mentioned in this regard, that the patients involved in these case reports had been treated unsuccessfully with various DMARDs (disease-modifying antirheumatic drugs, i.e., methotrexate, sulfasalazine, hydroxychloroquine, leflunomide) as well as TNFα inhibitors before the start of rituximab. Therefore, the population studied was very difficult-to-treat, a fact that should be taken into consideration while interpreting study results.

Another treatment option in the case of methotrexate/rituximab failure or adverse reactions is abatacept. There are two case reports demonstrating neutrophil normalization in rituximab-naive patients using intravenous or subcutaneous administration of abatacept (78, 79).

In two case reports, cortisone-free neutrophil normalization over a period of 2 years was achieved using hydroxychloroquine (HCQ). Interestingly, neutrophil counts correlated with HCQ metabolites in whole blood and whole blood levels below target were associated with recurrent neutropenia. The target level was defined using the sum of two HCQ metabolites, i.e., desethylchloroquine (DCQ) and desethylhydroxychloroquine (DHCQ), measured in whole blood (target level: DCQ + DHCQ >1,000 ng/mL) (80). To achieve this target level, markedly higher HCQ doses are necessary (up to 1,200 mg HCQ per day), which are otherwise unusual in rheumatology because of concerns regarding ocular toxicity.

There are a few case reports showing some efficacy of cyclosporin A as well. But in most cases, patients also received other immunosuppressants [e.g., hydroxychloroquine (81)] and methylprednisolone (4–6 mg per day) at the same time, making it hard to figure out efficacy of cyclosporin A alone. But at least, tapering of methylprednisolone was possible under cyclosporin A treatment (81–83).

Data on leflunomide are conflicting. While Talip et al. reported an increase in neutrophil counts and the possibility of stopping glucocorticoid treatment under leflunomide (starting with leflunomide 100 mg per day for 3 days, followed by 20 mg per day), other case reports did not show any significant efficacy (72, 84).

Until now, TNFα inhibitors like etanercept, infliximab and adalimumab have not shown any significant improvement in neutrophil counts in several case reports, although synovitis has been treated successfully with these agents (71, 72, 85, 86). In this context it should be noted, that immunosuppressants with proven efficacy in Felty’s syndrome should be used in any case, even if arthritis is well controlled by the current treatment. Thus, a change in treatment might be necessary (e.g., addition of methotrexate to an existing TNFα inhibitor therapy or switch to rituximab).

Regarding tocilizumab, there is just one case report showing efficacy in Felty’s syndrome (87) and concerning JAK (Janus kinase) inhibitors, there is no data yet. But JAK inhibitors might turn out to be a promising treatment option, as tofacitinib and upadacitinib improved neutropenia in patients with rheumatoid arthritis accompanied by T-LGL leukemia (88, 89). The studied population was difficult-to-treat and various treatments (including methotrexate, rituximab, cyclosporin A, cyclophosphamide) had failed before starting a JAK inhibitor. Tofacitinib was linked to apoptosis of T-LGLs *in vitro*, especially in the context of existing STAT3 mutations (88). As mentioned above, T-LGLs as well as STAT3 mutations might play a role in at least some cases of Felty’s syndrome too. Considering these common pathomechanisms, JAK inhibitors might prove useful in the treatment of Felty’s syndrome in the future.

Because it takes several weeks for immunosuppressants to have an effect, glucocorticoids and G-CSF are necessary as bridging. Glucocorticoids rapidly improve synovitis as well as neutropenia. Glucocorticoids increase neutrophils through acceleration of cell maturation, mobilization of neutrophils from the bone marrow and the marginal pool, reduction of leukocyte extravasation and by prolonging neutrophil survival via anti-apoptotic signaling (90).

However, in Felty’s syndrome, prednisolone doses ≥30 mg per day are often necessary to improve neutropenia significantly (25, 37) and doses below 7.5 mg per day usually fail to sustain stable neutrophil counts in the absence of additional immunosuppressive treatment. This is further complicated by the fact that some patients with Felty’s syndrome do not respond to glucocorticoids adequately (25, 37) and that glucocorticoids should be avoided in the presence of active infection. To improve neutrophil counts rapidly in the presence of infection, administration of G-CSF is useful. In patients with Felty’s syndrome, filgrastim and lenograstim were used in particular. G-CSF dosage and frequency of administration depend on neutrophil count. An ANC target level of >1,500/μL can be recommended. In a case series of Stanworth et al., frequency of administration varied between once a day and two times a week. Standard doses as well as reduced doses of filgrastim (300 μg and 150 μg respectively) and lenograstim (263 μg and 105 μg respectively) were used over a period of up to 3.5 years. Adverse reactions (severe arthralgia, cutaneous vasculitis) only occurred at the beginning of the standard dose regime, thus starting with reduced doses and a gradual dose escalation can be recommended (91). Overall, G-CSF therapy seems to be safe over a considerable period of time.

**Figure 1:**
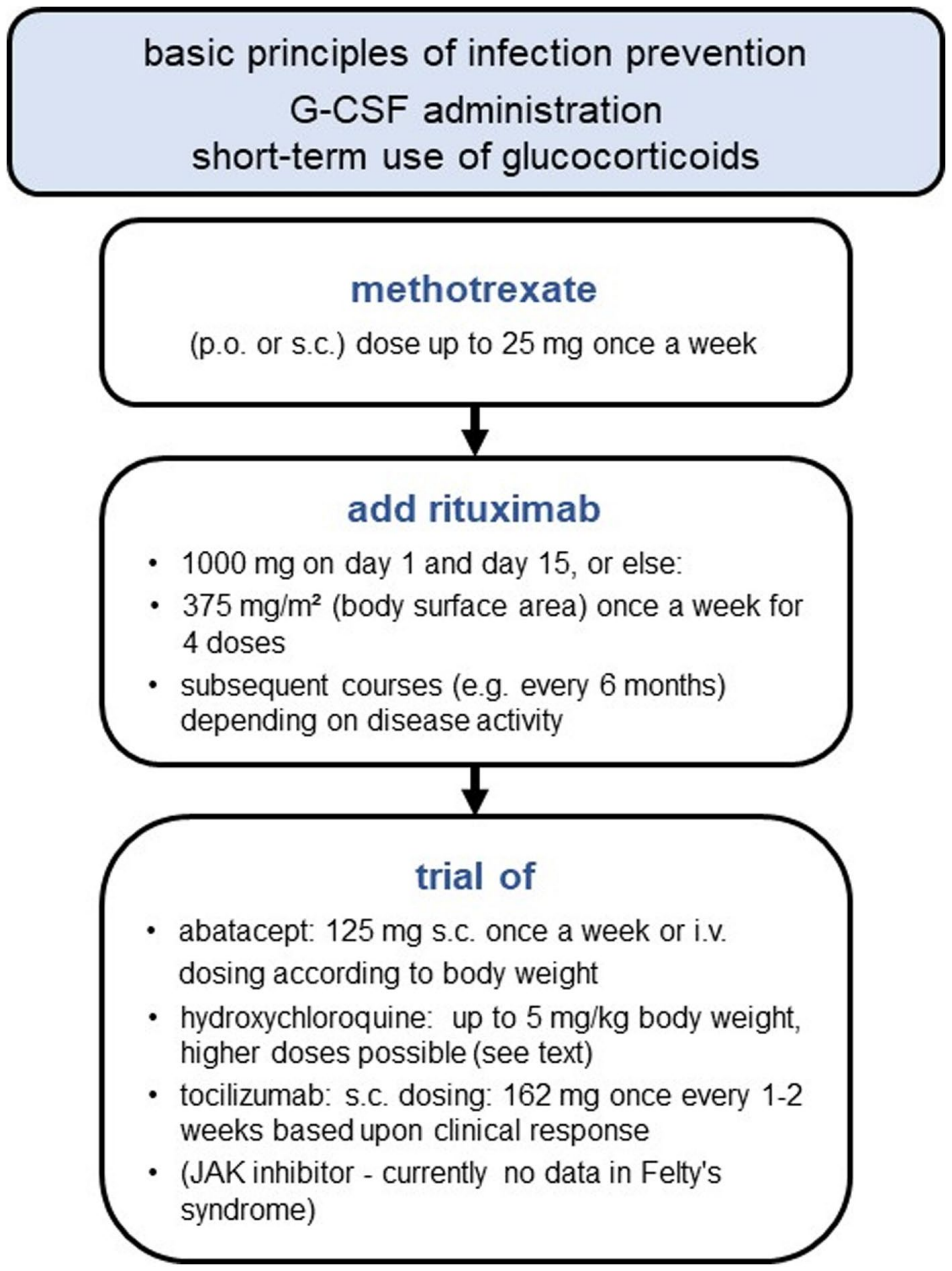
Stepwise approach for Felty’s syndrome treatment.

A treatment algorithm is depicted in Figure 1. Patients with Felty’s syndrome should also be informed about basic principles of infection prevention (Supplementary Table S4).

## Conclusion

Felty’s syndrome often comes along with diagnostic challenges, as there are many conditions/diseases with similar manifestations. To rule out malignancies, bone marrow examination is usually required. Although neutropenia with ensuing infections is still the main cause of death in Felty’s syndrome, other sequelae like non-cirrhotic portal hypertension leading to gastrointestinal bleeding as well as an increased lymphoma risk contribute to its morbidity and should be therefore kept in mind as well. Methotrexate and rituximab are still the mainstay of treatment and in most cases, Felty’s syndrome is sufficiently controlled by these drugs. But there are difficult-to-treat cases, necessitating new treatment options. Maybe JAK inhibitors might prove useful in the future, as there have been promising results in patients with T-LGL leukemia, which seems to share some pathomechanisms with Felty’s syndrome.

## Author contributions

CW did the literature research and wrote the article. VF had the idea and supervised the process. KS and AM revised the article and provided input and critical feedback. All authors contributed to the article and approved the submitted version.
