# Supplementary material for: Felty’s syndrome
Source: Front Med (Lausanne). 2023 Oct 17;10:1238405. doi: 10.3389/fmed.2023.1238405 (PMC10619942; doi:10.3389/fmed.2023.1238405)
Supplement: Supplementary file 1 [file Table_1.DOCX]

Supplementary table 1: Causes of splenomegaly

| **Infection**   - viral (e.g. EBV, CMV, HIV, hepatitis) - bacterial (e.g. typhus, brucellosis, leptospirosis, tuberculosis) - fungal (e.g. histoplasmosis) - parasitic (e.g. malaria, toxoplasmosis, leishmaniasis, trypanosomiasis, babesiosis) |
| --- |
| **Malignancy**   - lymphoma - acute and chronic leukemias - myeloproliferative neoplasms - metastases - some histiocytic disorders |
| **Metabolic diseases/storage diseases**   - mucopolysaccharidoses (e.g. Hurler syndrome) - sphingolipidoses (e.g. Gaucher disease, Niemann-Pick disease) - Tangier disease - amyloidosis |
| **Hemolytic anemia** (e.g. hemoglobinopathies, spherocytosis, autoimmune hemolytic anemia) |
| **Inflammation**   - autoimmune diseases (e.g. SLE, Felty’s syndrome) - sarcoidosis - hemophagocytic lymphohistiocytosis |
| **Congestive**   - liver cirrhosis - heart failure - obstruction of hepatic, portal, splenic veins |

Supplementary table 2: Causes of neutropenia

| - **Infection** (viral, bacterial, parasitic) - **Medication** (either dose-dependent or idiosyncratic) - **Malignancy** (e.g. lymphoproliferative disorders, myelodysplastic syndromes) - **Autoimmune diseases** (e.g. autoimmune neutropenia, SLE, Sjögren’s syndrome, Felty’s syndrome) - **Nutritional** (e.g. deficiencies of vitamin B12, folate, copper) - **Congenital** (e.g. cyclic neutropenia, Shwachman-Diamond syndrome) |
| --- |

Supplementary table 3: Overview of hematologic conditions/diseases included in the differential diagnosis of Felty’s syndrome (a: clinical features, b: serology, c: immunophenotype, d: clonality). Frequencies of clinical features reported in different studies are put in parentheses.

|  | **Felty’s syndrome** | **reactive LGL lymphocytosis** | **T-LGL leukemia** | **HSTL** |
| --- | --- | --- | --- | --- |
| **a** | erosive polyarthritis  rheumatoid nodules  (71-82%)  splenomegaly (> 90%)  neutropenia (100%)  anemia (79-100%)  thrombopenia (15-48%) | features of underlying rheumatic disease possible (e.g. rheumatoid arthritis, SLE) | | |
|  |  | --- | B symptoms (7-12%)  neutropenia (52-84%)  anemia (24-89%)  thrombopenia (19-36%)  splenomegaly (19-50%)  hepatomegaly (1-32%)  lymphadenopathy  (1-13%) | B symptoms (67-80%)  neutropenia (36-57%)  anemia (57-73%)  thrombopenia (64-95%)  splenomegaly (100%)  hepatomegaly (40-71%)  lymphadenopathy  (< 13%) |
| **b** | RF positive (90-100%)  ACPA positive (96%)  ANA positive (55-84%) | depends on underlying rheumatic disease | | |
| **c** | reactive T-LGL and/or NK-LGL lymphocytosis possible | T-LGL: mostly CD3^+^CD8^+^CD16^+^CD57^+^ with α/β TCR (less often γ/δ TCR)  NK-LGL: mostly  CD3^-^CD4^-^CD8^-^CD16^+^CD56^+^CD57^-^ | mostly CD3^+^CD8^+^CD16^+^CD57^+^ with α/β TCR (less often γ/δ TCR) | mostly  CD4^-^CD8^-^CD56^+/-^ with γ/δ TCR (less often α/β TCR) |
| **d** | if reactive LGL lymphocytosis is present: mostly poly- or oligoclonal, rarely monoclonal | mostly poly- or oligoclonal, rarely monoclonal | monoclonal | monoclonal |

Supplementary table 4: Basic principles of infection prevention

| - **Hand hygiene**: wash your hands regularly with soap and water and use hand sanitizer - **Dental hygiene**: brush your teeth with fluoride toothpaste and attend your dentist regularly - **Food hygiene**: avoid eating uncooked meat/fish/shellfish or unpasteurized dairy products, only use clean water - **Pet hygiene**: avoid contact with droppings, keep pets/animals away from your food, rapidly disinfect bite and scratch wounds, let your pet be checked by a veterinarian regularly - **Vaccination**: update your vaccination status according to national vaccination recommendations, get recommended travel vaccinations before travelling |
| --- |
